# The usefulness of the sum of rotation parameter in scoliosis screening

**DOI:** 10.1186/1748-7161-7-S1-O59

**Published:** 2012-01-27

**Authors:** J Chowańska, T Kotwicki

**Affiliations:** 1 Rehasport Clinic, Poznań, Poland; Spine Disorders Unit, Department of Pediatric Orthopedics and Traumatology, University of Medical Sciences, ul. Poznan, Poland; 2 Spine Disorders Unit, Department of Pediatric Orthopedics and Traumatology, University of Medical Sciences, Poznan, Poland

## Purpose of the study

It was to assess the usefulness of the Sum of Rotation parameter in scoliosis screening.

## Background

The Sum of Rotation parameter (SR) represents the sum of absolute values of angles of trunk rotation (ATRs) measured with a scoliometer on three spinal levels: proximal thoracic, main thoracic and thoracolumbar/lumbar. This parameter is useful in following-up the patient during conservative scoliosis management, because it documents the global rotational trunk deformity [1-4].

## Materials and methods

In a cohort of 996 school girls, aged 11.0 ± 1.0 years, range 9 to13, the average, standard deviation, minimum and maximum values of SR were calculated separately for three distinct subgroups: (a) ATR: 0°- 3°, (b) ATR 4° to 6° and (c) ATR ≥ 7°.

## Results

The SR value was: (a) 0.9 ± 1.2, range 0.0° to 6.0°, N=870, (b) 5.5 ± 1.9, range 4.0° to 17°, N= 94, (c) 10.2 ± 3.3, range 7.0° to 20.0°, N= 32. Normal children with important SR values were identified (SR = 2°+2°+2° = 6°).

## Conclusions

The use of SR parameter seems not reveal additional information in scoliosis screening comparing to simple ATR measurement. Moreover, a risk of bias is present if the cut-off criterion of 7° is applied.
